# Editorial: Caveolins in inflammation, infections, and disease

**DOI:** 10.3389/fimmu.2024.1387191

**Published:** 2024-03-05

**Authors:** Stanley Hoffman, Clarissa M. Maya-Monteiro, Cecilia J. G. de Almeida

**Affiliations:** ^1^ Division of Rheumatology and Immunology, Medical University of South Carolina, Charleston, SC, United States; ^2^ Laboratory of Immunopharmacology, Oswaldo Cruz Institute, Oswaldo Cruz Foundation (Fiocruz), Rio de Janeiro, Rio de Janeiro, Brazil

**Keywords:** caveolin, inflammation, infection, disease, sepsis, fibrosis, cancer, metabolism

Caveolins are essential structural components of caveolae, plasma membrane invaginations involved in transcytosis and mechanoprotection. Caveolins are also found outside caveolae, in the plasma membrane or other organelles, and regulate many cellular processes, such as lipid metabolism, proliferation, and autophagy. Nevertheless, caveolins are not essential for life as caveolin knockout mice are viable.

Caveolin loss or mutation is associated with diseases including metabolic disorders, cancer, and fibrosis. Among their many activities, caveolins regulate inflammation and metabolism, thus underlying several pathologies (**Figure 1**). Articles in this series address many pathologies in which caveolin-1 (Cav-1) is involved, the multiple biological processes it regulates, and the multiple underlying molecular mechanisms involving Cav-1.

**Figure 1:**
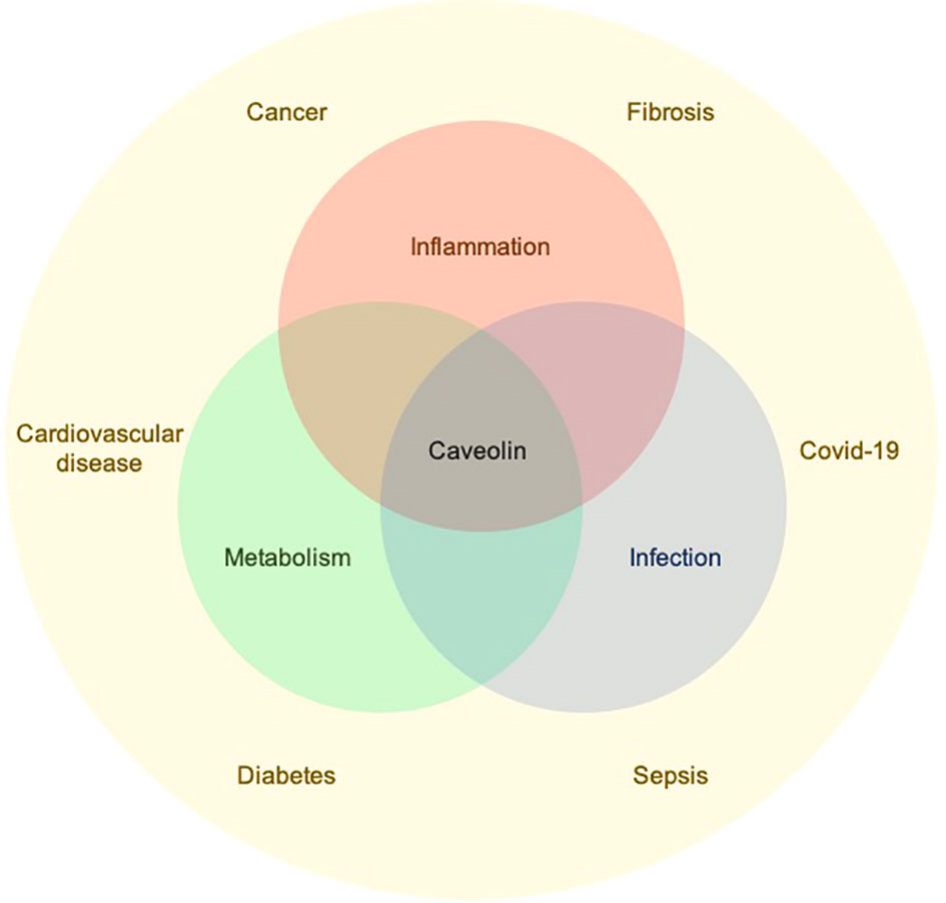
Caveolins regulate inflammation, metabolism, and infection which underlie the development of several pathological conditions.

King et al. report on Cav-1 in breast tumor growth. This is the first report that Cav-1 may underly cancer health disparities. It is well known that low Cav-1 in the tumor stroma is correlated with poor patient outcomes. They report that the Cav-1 level in the breast cancer stroma of black women is lower than in white women. They also provide a novel mechanism for Cav-1 protein regulation. MiRNA miR-510-5p is overexpressed in breast cancer patients, particularly African Americans. Epithelially-produced MiR-510-5p downregulates fibroblast Cav-1, resulting in fibroblast activation. In summary, the miR-510-5p – Cav-1 axis plays a central role in controlling tumor growth via tumor-stromal interactions and in cancer health disparities.

Takamura and Yamaguchi present a review on Cav-1 in skin diseases. Cav-1 is expressed in multiple cell types. The most common alteration is Cav-1 loss which occurs in several cell types including keratinocytes and monocytes in psoriasis; fibroblasts, adipocytes, monocytes, and mesenchymal stem cells in scleroderma; and fibroblasts in scar tissue and keloids. In all cases, Cav-1 loss leads to cellular activation. Cav-1 as a therapeutic target is discussed, particularly in terms of the feasibility and beneficial effects of treating cells or mice with the Cav-1 scaffolding domain (CSD). CSD is the active site of Cav-1 for inhibiting multiple kinases and other enzymes involved in signaling. CSD has many beneficial effects in mouse model systems for psoriasis and scleroderma and in cultured fibroblasts from scar tissue and keloids. The beneficial effects of full-length (20 amino acids) CSD are retained by three independent 8-amino acid CSD subregions. In summary, skin diseases are particularly suitable for demonstrating the roles of Cav-1 in disease and the beneficial effects of targeting Cav-1 deficiency using versions of CSD to mimic Cav-1 function.

Xia et al. present a review of the importance of caveolins in the pathogenesis of diabetic cardiomyopathy (DCM). They carefully review DCM pathogenesis and highlight the roles of Cav-1 and Cav-3. DCM progression involves multiple factors, including inflammation, hyperglycemia, and oxidative stress that evolve into endothelial dysfunction, myocardial cell death, and heart failure. Cav-1 and Cav-3 are important players in DCM onset. Deficiency of either protein leads to DCM, possibly by affecting insulin signaling and resistance. They also draw parallels between the role of caveolins and adiponectin signaling in tissue homeostasis. They propose a combination of the caveolin and adiponectin pathways as targets for DCM treatment.

The review by Lannes-Costa et al. addresses how various Cav-1 activities in distinct cell types control the inflammatory response and sepsis, a severe condition resulting from a systemic response to infection leading to tissue damage, organ failure, and death. Cav-1 is important for infection by pathogens that exploit caveolae to enter cells and escape lysosomal degradation. Cav-1 coordinates the cellular response to infectious and inflammatory stimuli in several ways. It organizes the plasma membrane lipid environment, affecting the location of receptors and their connection with the cortical cytoskeleton. It impacts the host response through its ability to interact with and regulate pathogen recognition receptors and downstream signaling proteins. The review also emphasizes caveolin-dependent activities modulating the host response during sepsis in the lungs and brain. Cav-1 is highly expressed in various cell types of the lungs including lung epithelial cells, vascular cells, and airway smooth muscle cells. Interestingly, Cav-1 expression is diminished in various lung cell types in distinct lung pathologies. Restoring Cav-1 expression or introducing CSD peptides alleviates the severity of these pathologies. In sepsis and neuroinflammation, Cav-1 regulates blood-brain barrier permeability by controlling transcytosis, the integrity of tight junctions, nitric oxide production, and other mechanisms critical for fluid extravasation and leukocyte recruitment. Finally, they discuss targeting Cav-1 to discover urgently needed new therapies for sepsis.

Two studies demonstrate new interesting roles for Cav-1 in response to infection:

Sivanantham et al. indicate that Cav-1 regulates the inflammatory response of the host to infection. They show that *Escherichia coli*-derived outer membrane vesicles (OMVs) activate an M1 profile in macrophages and that, in the absence of Cav-1, this response is even more intense. Loss of Cav-1 expression further enhances Il-1β and ROS production, but does not alter macrophage migration, indicating that Cav-1 modulates only certain aspects of macrophage activation. Moreover, the effects of Cav-1 absence differ between mouse macrophage and human THP-1-derived macrophages. Whereas loss of Cav-1 increases inflammatory mediators in mouse primary macrophages, it has no effect in THP-1-derived human macrophages. Furthermore, OMVs induce the expression of various toll-like receptors (TLRs) while diminishing Cav-1 expression. In the absence of Cav-1, TLR expression further increased. These results provide novel insights regarding the limitation of the pro-inflammatory response by Cav-1 when immune cells are stimulated by OMV.

Batori et al. explore another role for Cav-1 in modulating infection and inflammation. Pneumolysin (PLY) is a primary virulence factor of *Streptococcus pneumonia* responsible for endothelial cell (EC) pore formation and alterations in intracellular Ca2+ with consequent endothelial barrier dysfunction. The authors show that Cav-1-deficient EC are more sensitive to PLY-induced disruption. Both Cav-1 overexpression and treatment with CSD attenuated the PLY effect in Cav-1-deficient EC. They further show that Cav-1’s capacity to regulate endocytosis of damaged membranes is important in maintaining cell membrane integrity and protecting the endothelial barrier against PLY.

Finally, in line with the role of caveolins in infections, Cesar-Silva et al., present a review of the role of caveolins and caveolae in coronavirus infection, as well as of other lipid compartments, such as lipid rafts and lipid bodies. In addition, the review discusses how lipid metabolism behaves during coronavirus infection and presents the various therapeutic strategies that explore the manipulation of lipid metabolism to combat these infections. To systematically present the effect of these drugs on infection by diverse coronaviruses, the authors organized a table with an extensive list of substances that alter lipid metabolism with their corresponding effects on infection in *in vitro* and *in vivo* experiments, as well as in clinical trials.

## Author contributions

CA: Writing – review & editing, Writing – original draft, Conceptualization. CM-M: Writing – review & editing, Writing – original draft, Conceptualization. SH: Writing – review & editing, Writing – original draft, Conceptualization.

